# Data on biology and demographic parameters of the *Aedes albopictus* from dengue outbreaks in Klang Valley, Malaysia

**DOI:** 10.1016/j.dib.2020.105882

**Published:** 2020-06-20

**Authors:** Nazri Che Dom, Ibrahim Ahmed Alhothily, Siti Nazrina Camalxaman, Sharifah Norkhadijah Syed Ismail

**Affiliations:** a Centre of Environmental Health & Safety, Faculty of Health Sciences, Universiti Teknologi MARA, UiTM Cawangan Selangor, 42300 Puncak Alam, Selangor, Malaysia; b Integrated Mosquito Research Group, Faculty of Health Sciences, Universiti Teknologi MARA, UiTM Cawangan Selangor, 42300 Puncak Alam, Selangor, Malaysia; c Department of Occupational Health and Safety, Faculty of Medicine and Health Sciences, Universiti Putra Malaysia, 43400 Serdang, Selangor, Malaysia

**Keywords:** Demographic parameters, *Aedes albopictus*, Dengue areas, Malaysia

## Abstract

In this article, data on the demographic parameters of the *Aedes albopictus* were collected from those areas in Shah Alam, Malaysia that had experienced a dengue outbreak. The surveys were conducted from March to December 2017. The eggs of the *Ae. albopictus* were collected using ovitraps, and were analysed based on the demographic parameters in a controlled environment in an insectarium. The data were comprised of four types of biological information on the life demographic parameters of the *Ae. albopictus* that were monitored based on specific localities. The data were inferred information regarding egg productivity (*n*), egg development (%), immature development (days), and survivorship (days).

**Table of Specifications**

**Table:** 

**Subject**	Biology
**Specific subject area**	Bioscience and Biodiversity, Entomology
**Type of data**	Tables GIS maps
**How data were acquired**	This study was performed using a field strain of the *Ae. albopictus* that was originally collected from twenty residential areas in the central zone of Shah Alam. This research applied fieldwork with a cross-sectional design to investigate the demographic parameters of the *Ae. albopictus*. The evaluation of the *Aedes albopictus* was conducted in a controlled environment in an insectarium.
**Data format**	Raw Semi-analysed
**Parameters for data collection**	Biological information was obtained on life demographic parameters, namely, (i) immature development, (ii) adult survival, (iii) gonotrophic cycle, and (iv) fecundity of the adult *Ae. albopictus*. The adult mosquitos were monitored according to the set localities.
**Description of data collection**	Descriptive demographic parameters were used to describe the field strain of the *Ae. albopictus* collected from residential areas in Shah Alam. The eggs of the *Ae. albopictus* that had been collected were colonised in the Vector Control Research Laboratory (at a temperature of 28 ± 2 °C with 75% to 85% relative humidity) using standard protocols adopted from the “Manual for Mosquito Rearing and Experimental Techniques” [1, 2].
**Data source location**	The entomological survey was conducted in twenty localities in the central zone of Shah Alam, Malaysia. The GPS coordinates used to collect the field strains were as follows:
**Data accessibility**	All the raw data are available in the article.

**Value of the Data**

• Data on the life parameters of the *Ae. albopictus* mosquito are beneficial for the scientific community to understand the population dynamics of a species within their habitat. This is important for interpreting the life demographics, which include the survival, development and reproductive system of a population under various conditions.
• Data on the development rate of the *Ae. albopictus* in dengue outbreak areas are necessary for the scientific community to estimate the ability of the vector in transmitting the disease.
• Other scientists can benefit from the data when dealing with studies related to mosquito ecology. Furthermore, the survival of mosquitoes plays a pivotal role in disease transmission.
• The data can be used to understand the demographic parameters of the mosquito to strengthen existing vector control programmes. The data from the research that was conducted in dengue outbreak areas in Malaysia in 2017 offer information on the demographic parameters of the *Ae. albopictus*.

## 1 Data description

**Fig. 1:**
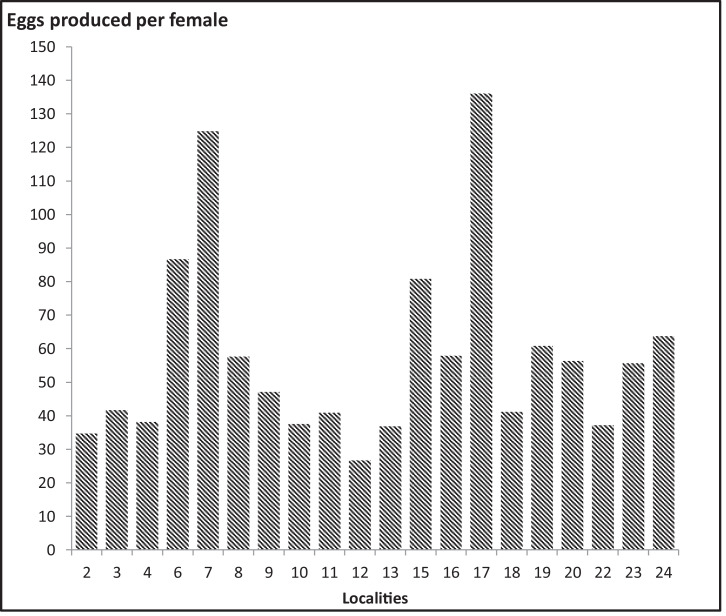
Average number of eggs laid by female *Ae. albopictus* at twenty localities in the central zone of Shah Alam.

The present study was undertaken in twenty districts in the central zone of Shah Alam. Out of a total of 600 ovitraps that were placed in the field during the study period, 371 positive ovitraps (66.21%) were collected, while a total of 10,606 eggs were obtained (Table 1). To summarize the population parameter of the *Ae. albopictus* in the central zone of Shah Alam, the following factors were analysed: (i) egg development, (ii) immature development, (iii) adult survivorships, (iv) female fecundity, and (v) gonotrophic cycle. The study was developed under controlled laboratory conditions (at a temperature of 28 ± 2 °C with 75% to 85% relative humidity). Tables 2 and 3 show the descriptive population parameters of the *Ae. albopictus* at the immature and adult stages, respectively, while the biological data on the gonotrophic cycle and fecundity are tabulated in Table 4. Based on the outcome, 18 localities (78.2%) in the central zone showed a developmental time of between 6 and 9 days. In terms of adult longevity, 11 localities (47.8%) were observed to have a longer longevity period, where the adult was able to survive for more than 54 days. The fecundity, gonotrophic cycle, and generations produced were also observed in order to determine the biology and demographic parameters of DF. The highest fecundity was recorded in Section 17, with a total of 3402 eggs and seven gonotrophic cycles until the adult mosquitoes died. The locality with the lowest fecundity was Section 12, with a total of 667 eggs and only 6 gonotrophic cycles. On average, a female *Ae. albopictus* in these areas laid between 26.68 to 136.08 eggs (Fig. 1).

**Table 1:** Positive ovitrap index (POI) and mean eggs per trap (MET) collected from twenty localities in the central zone of Shah Alam, Malaysia.

Locality	Ovitrap placement (a)	Number of recovered ovitrap (b)	Number of positive ovitrap (c)	Positive ovitrap index (POI) (%)	Number of eggs (d)	Mean eggs per trap (MET±SD)
S2	30	28	21	75.00	482	22.95±7.64
S3	30	27	18	66.70	477	26.50±17.00
S4	30	23	20	86.95	407	20.35±9.60
S6	30	29	18	62.07	523	29.06±10.00
S7	30	29	25	86.21	1023	41.68±11.92
S8	30	27	15	55.56	321	21.40±7.01
S9	30	23	15	65.21	413	27.53±19.23
S10	30	30	19	63.33	487	25.63±9.56
S11	30	29	15	51.72	457	30.47±8.18
S12	30	29	16	55.17	317	19.81±14.21
S13	30	29	13	44.83	260	20.00±5.15
S15	30	26	20	76.92	716	35.80±8.03
S16	30	29	17	58.62	366	21.53±8.01
S17	30	28	21	75.00	974	38.96±17.18
S18	30	30	22	73.33	764	34.73±6.08
S19	30	30	20	66.67	740	37.00±6.94
S20	30	30	21	70.00	840	40.00±8.72
S22	30	29	17	58.62	301	17.71±8.21
S23	30	24	14	58.33	170	12.14±5.67
S24	30	27	20	74.07	568	28.40±9.14
Total	**600**	**556**	**371**	**66.21**	**10,606**	**28.58±6.38**

Note: The positive ovitrap index (POI) was calculated by dividing the number of positive ovitraps (c) with the total number of recovered ovitraps (b) and multiplying with 100. The mean eggs per trap were calculated by dividing the total number of eggs collected (d) with the number of positive ovitraps (c).

**Table 2:** Descriptive population parameters of the *Ae. albopictus* (immature stage) at study sites within residential areas in the central zone of Shah Alam, Malaysia.

Locality	Development rate, (*n*)				Developmental day of immature, F_0_ (Mean, days)				Population performance	
	Eggs	Larvae	Pupae	Adult	Eggs	Larvae	Pupae	Adult	Hatching rate (%)	Mortality rate (%)
S2	482	420	307	297	2	4	1	7	87.14	29.28
S3	477	350	320	314	1	5	2	8	73.37	10.28
S4	407	341	227	208	3	4	2	9	83.78	39.00
S6	523	476	381	355	3	5	1	9	91.01	25.42
S7	1023	701	587	507	2	4	1	7	68.52	27.67
S8	321	283	192	183	4	5	2	11	88.16	35.33
S9	413	300	270	257	1	5	2	8	72.64	14.33
S10	487	421	327	302	1	5	2	8	86.45	28.27
S11	457	399	313	284	3	4	1	8	87.31	28.82
S12	317	210	151	139	2	6	2	10	66.24	33.81
S13	260	215	137	128	3	6	1	10	82.69	40.47
S15	716	579	404	385	2	5	2	9	80.86	33.51
S16	366	247	210	201	2	5	2	9	67.49	18.62
S17	974	866	723	704	1	5	1	7	88.91	18.71
S18	764	749	603	586	3	5	2	10	98.04	21.76
S19	740	687	555	516	2	4	2	8	92.84	24.89
S20	840	750	616	566	4	4	1	9	89.29	24.53
S22	301	283	199	175	2	4	1	7	94.01	38.16
S23	170	147	92	85	3	7	1	11	86.47	42.17
S24	568	412	324	295	4	5	2	11	72.53	28.40

Note: The sites are described as *S* = Section in residential areas in the central zone of Shah Alam, Malaysia. The annotation, (*n)*, represents the number of eggs, larvae, pupae and adults of the *Ae. albopictus*.

**Table 3:** Descriptive population parameters of the *Ae. albopictus* (adult stage) at study sites within residential areas in the central zone of Shah Alam, Malaysia.

Locality	Emerged adult, F0, (*n)*				Survivorship of adult, lx (days)		
	Total	Male	Female	ratio	Male	Female	Average
S2	297	106	191	1.0:1.8	57	67	62
S3	314	235	79	3.0:1.0	61	74	67.5
S4	208	83	125	1.0:1.5	38	43	40.5
S6	355	213	142	1.5:1.0	65	79	72
S7	507	195	312	1.0:1.6	64	73	68.5
S8	183	96	87	1.1:1.0	41	57	49
S9	257	122	135	1.0:1.1	48	66	57
S10	302	100	202	1.0:2.0	33	45	39
S11	284	86	198	1.0:2.3	39	45	42
S12	139	76	63	1.1:0.9	29	41	35
S13	128	76	52	1.5:1.0	29	43	36
S15	385	148	237	1.0:1.6	46	68	57
S16	201	74	127	1.0:1.7	50	57	53.5
S17	704	287	417	1.1:1.6	70	77	73.5
S18	586	320	266	1.2:1.0	37	49	43
S19	516	297	219	1.5:1.1	50	59	54.5
S20	566	271	295	1.1:1.2	47	51	49
S22	175	87	88	1.0:1.0	55	70	62.5
S23	85	46	39	1.2:1.0	52	68	60
S24	295	134	161	1.0:1.2	60	69	64.5

Note: Identification and status of the *Ae. albopictus* follow the descriptions listed by the CDC, whereby the F0 (field strain) was subjected to mass rearing. The process was conducted in a controlled environment in an insectarium at a temperature of 28 ± 2 °C with 75 ± 10% humidity and a photoperiod of 14:10 h of dark:light cycles. The annotation in brackets (*n*) represents the number of adult *Ae. albopictus* that were utilised to obtain the desired measurements. Additionally, the survivorship of the adults (lx) was monitored and recorded daily (days).

**Table 4:** The unprocessed data on the fecundity, gonotrophic cycle, and generation of offspring of the *Ae. albopictus* strain collected from study sites (25 males: 25 females).

Locality	Gonotrophic cycle of F0 (Total eggs produced)									Total eggs (*n*)	No. eggs per ♀	Total cycle	Mean eggs per cycle
	G1	G2	G3	G4	G5	G6	G7	G8	G9				
S2	125	368	214	117	32	14	0	0	0	870	34.80	6	145.00
S3	41	186	100	332	147	123	50	39	25	1043	41.72	9	115.89
S4	127	350	317	108	30	21	0	0	0	953	38.12	6	158.83
S6	56	437	625	546	324	89	39	21	29	2166	86.64	9	240.67
S7	71	1050	813	651	318	95	77	45	0	3120	124.8	8	390.00
S8	108	435	516	238	94	31	19	0	0	1441	57.64	6	205.86
S9	388	547	101	63	58	21	0	0	0	1178	47.12	7	196.33
S10	100	174	235	214	157	42	18	0	0	940	37.60	6	134.29
S11	200	325	204	175	87	32	0	0	0	1023	40.92	6	170.50
S12	97	201	189	116	25	39	0	0	0	667	26.68	7	111.17
S13	108	186	201	219	133	56	18	0	0	921	36.84	6	131.57
S15	100	432	475	387	358	104	109	32	25	2022	80.88	7	224.67
S16	70	300	357	270	210	199	40	0	0	1446	57.84	7	206.57
S17	71	1250	854	600	318	187	77	45	0	3402	136.08	8	425.25
S18	10	87	332	368	120	88	25	0	0	1030	41.2	7	147.14
S19	64	142	589	432	129	78	63	25	0	1522	60.88	8	190.25
S20	100	430	515	226	80	32	24	0	0	1407	56.28	7	201.00
S22	100	175	265	217	125	36	12	0	0	930	37.20	7	132.86
S23	114	1007	108	116	30	15	0	0	0	1390	55.60	6	231.67
S24	207	289	299	346	265	104	50	35	0	1595	63.80	8	199.38

Notes: A gonotrophic cycle is the period of time from the blood supply to the oviposition. The gonotrophic cycle, which is represented by the total number of eggs produced per cycle, is denoted by G. Descriptions of the measurements are abbreviated as follows: *n* = total number of eggs produced, and ♀ = No. of eggs produced by a single female mosquito.

The incidence rate (IR) was used to compare the biological parameters of the *Ae. albopictus* in high and low-risk localities in the central zone of Shah Alam. This IR data for residential areas were categorized into two groups; namely (i) high-IR (>20 cases in a population of 1000), and (ii) low-IR (<20 cases in a population of 1000) areas. An independent *t-test* analysis was used to determine if there was a significant difference between the biological parameters of the *Ae. albopictus* in high and low-IR areas. Generally, most of the data recorded was greater in the high-IR areas compared to the low-IR areas, except for the immature development time (larvae to pupae, and the total development time) (Table 5). There was a significant difference (*p* = 0.03) in terms of the number of eggs produced by the females in high-IR (*M* = 73.27 ± 15.69 eggs) and in low-IR (*M* = 45.75 ± 11.43 eggs) areas.

**Table 5:** Table of life attributes of the *Ae. albopictus* mosquito in Shah Alam based on the incidence rate (IR).

Attribute	High IR		Low IR		*p*-value
	Mean ± SD	Range	Mean ± SD	Range	
**Female fecundity (egg productivity)**	73.27±15.69	36.84–136.08	45.75±11.43	26.68–60.88	0.03
**Egg development (%)**					
** Hatchability**	83.49±17.00	68.52–94.01	82.39±17.94	66.24–98.04	0.80
** Mortality**	31.42±7.40	18.71–42.17	25.51±7.63	10.28–39	0.14
**Immature development (days)**					
** Eggs to Larvae**	5.11±1.42	4–7	4.64±1.53	4–6	0.20
** Larvae to Pupae**	1.33±2.15	1–2	1.73±1.98	1–2	0.09
** Development time**	8.78±1.35	7–11	8.82±1.42	7–11	0.95
**Adult survivorships (days)**					
** Male**	52.67±7.44	29–70	45.18±8.69	29–61	0.18
** Female**	65.78±9.64	43–79	55.36±12.23	41–74	0.06
** Average**	59.22±8.45	36–73.5	50.27±10.43	35–67.5	0.10

Note: The descriptive analysis of the life attributes of the *Ae. albopictus* is based on the incidence rate (IR) of DF cases in 2017, which were categorized into high IR (>20 cases in a population of 1000) and low IR (<20 cases in a population of 1000). The *p*-values generated from the *t-test* analysis represent the significant difference between the attributes of the *Ae. albopictus* in high and low-IR areas.

## 2 Experimental design, materials, and methods

### 2.1 Study site

**Fig. 2:**
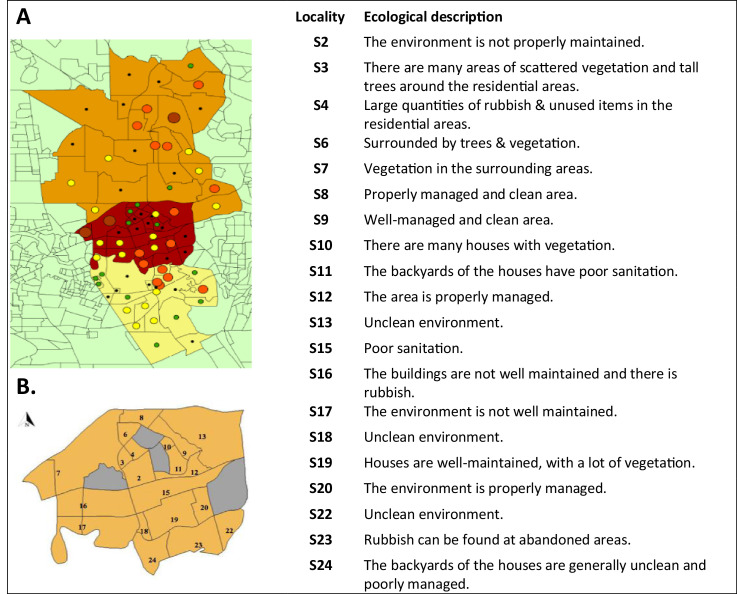
Cumulative distribution map for 2012 to 2017 with respect to the number of DF cases in Shah Alam. [A] Shah Alam is highlighted in different colours; orange: northern zone (NZ), brown: central zone (CZ), and yellow: southern zone (SZ). [B] Ecological description of the central Shah Alam area. Distribution map in terms of the number of DF cases in Shah Alam denoted by different colour codes; Dark brown: Very high; Orange: High; Yellow: Medium; Green: Low; and Black: Very low risk areas. .

The entomological survey was conducted in localities in the central zone of Shah Alam located approximately 25 km from the city of Kuala Lumpur at 3°05′48.74″ N 101°33′02.39″ E to 2°58′22.93″ N 101°44′ 39.69″ E altitude (Fig. 2). Based on the dengue surveillance data, this area experiences a large dengue outbreak every year. In this study, twenty residential localities were explored for the surveillance activities, and their surrounding environment was observed. The ecological conditions in the studied localities are summarized in Fig. 2. It was generally observed that this area had an intensely green landscape and vegetation.

### 2.2 Sampling and laboratory evaluation

**Fig. 3:**
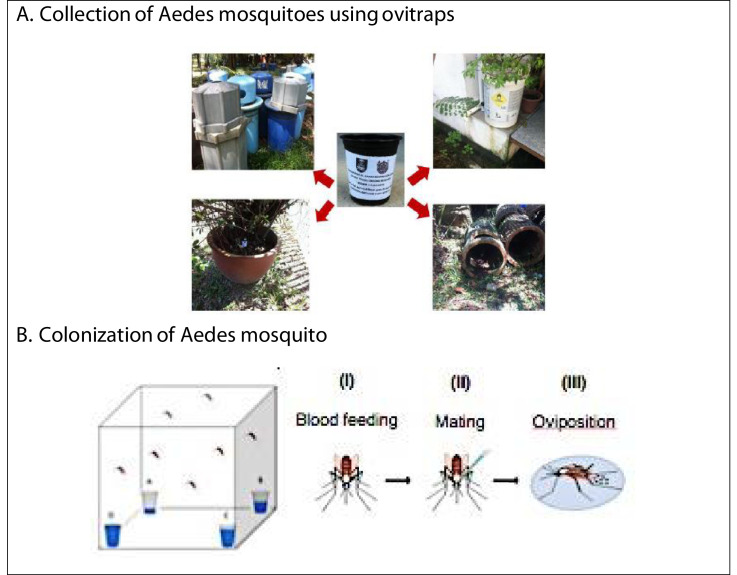
Sampling and laboratory evaluation; (A) Placement of ovitraps based on several factors; (i) near adult resting sites, (ii) in complete shade, away from the weather and human interference, (iii) in direct line of sight, (iv) near to other breeding containers, and (v) close to the ground. (B) Schematic representation of mass rearing activity in a 12:12 light:dark photoperiod.

The study was performed using a strain of the *Ae. albopictus* originally collected in Shah Alam from March to December 2017. The eggs were collected using ovitraps, which were essentially plastic containers filled with 150 ml distilled water, and with a paddle made of wooden hardboard (8 cm x 2 cm) for oviposition. A total of 20 ovitraps were deployed to each locality, and these were placed in habitats suitable for the breeding of mosquitos, such as (i) near adult resting sites; (ii) in complete shade, protected from the weather and human interference; (iii) in direct line of sight; (iv) near to other breeding containers; and (v) close to the ground (24). After four consecutive days of export, the ovitraps were collected back [3] (Fig. 3A). At this stage, the paddles inside the ovitraps were transferred into airtight plastic containers (13 cm × 6 cm). Meanwhile, the ovitraps were tightly covered with lids and brought back to the laboratory for the colonization of the mosquitos and enumeration of the eggs. All the collected ovitraps were labelled according to their prescribed localities. During the pre-experimental work, the paddles were first dried at a temperature of 28 °C to facilitate the egg-counting process.

In order to produce the F1 generation, the F_0_ (field strain) underwent mass rearing. The process was conducted in a controlled environment in an insectarium at a temperature of 28 ± 2 °C with 75 ± 10% humidity, and a photoperiod of 14:10 h of dark:light cycles. The eggs collected from each locality were hosted separately by placing them in water for one hour. First, the larval instars were transferred to plastic traps (29 × 23 × 6 cm) filled with dechlorinated tap water, and were monitored until adulthood. The larvae were fed daily with pupal larval food. The rate of development (days) of the eggs to the larval stage, and from the larval to the pupal stage, and finally, to immature mosquitoes, was observed and recorded accordingly [4].

The pupae that emerged were separated from the rest of the larvae and placed in a new container, which was put inside a standard rearing cage (30 cm x 30 cm x 30 cm) until the emergence of the adult mosquitoes. At the adult stage, the mosquitoes were separated according to their sex Then, 25 male and 25 female mosquitoes (ratio 1:1) were transferred into a container that was placed in a smaller cage (15 cm x 15 cm x 15 cm) using an insect separator, and this was inspected for the emergence of adult mosquitoes. A universal bottle was stuffed with cotton wool and filled with 10% sucrose solution to feed the mosquitoes. The universal bottle was placed in the cage and was refilled from time to time. After day 5 of the emergence of the adult mosquitoes, a blood meal was given by placing a lab rat in a confined cage for the development of the eggs in the female mosquitoes [5]. Following this, a round black plastic container was filled with dechlorinated water, and folded filter paper was placed on top to serve as an oviposition substrate. The moistened folded filter paper cone was changed daily until no eggs were deposited on it (Fig. 3B). The eggs, which had been placed in the containers with filter paper, were air-dried and the number of eggs was enumerated. The fecundity, gonotrophic cycle, and survival of the adult *Ae. albopictus* mosquitoes were monitored and recorded daily.

### 2.3 Data analysis and management

The present study was undertaken in twenty districts in the central zone of Shah Alam. In order to summarize the demographic parameters of the *Ae. albopictus,* a table analysis was conducted. The incidence rate (IR) of DF cases in 2017 was used to compare the demographic parameters in high and low-risk areas in the central zone of Shah Alam. For a descriptive analysis of the IR based on the number of DF cases in 2017, eleven sections (*n* = 11) were categorized as high-IR (>20 cases in a population of 1000) and nine sections (*n* = 9) were categorized as low-IR (<20 cases in a population of 1000) areas. Since the mode number for the incidence rate was 20.42, therefore, areas that recorded more than 20 cases were considered as high-incidence rate areas, while those that recorded less than 20 cases were considered as low-incidence rate areas. An independent *t-test* analysis was used to determine if there was a significant difference between the attributes of the *Ae. albopictus* in terms of the demographic parameters in the high and low-IR areas.

## Declaration of Competing Interest

The authors declare that they have no known competing financial interests or personal relationships, which have, or could be perceived to have, influenced the work reported in this article.

## Appendix Supplementary materials

Image, application 1
